# The coming of the Greeks to Provence and Corsica: Y-chromosome models of archaic Greek colonization of the western Mediterranean

**DOI:** 10.1186/1471-2148-11-69

**Published:** 2011-03-14

**Authors:** Roy J King, Julie Di Cristofaro, Anastasia Kouvatsi, Costas Triantaphyllidis, Walter Scheidel, Natalie M Myres, Alice A Lin, Alexandre Eissautier, Michael Mitchell, Didier Binder, Ornella Semino, Andrea Novelletto, Peter A Underhill, Jacques Chiaroni

**Affiliations:** 1 Department of Psychiatry and Behavioral Sciences, Stanford University School of Medicine, Stanford, CA 94305, USA; 2 Unité Mixte de Recherche 6578, Biocultural Anthropology, Centre National de la Recherche Scientifique, Etablissement Français du S ang and Université Faculté de Médecine - Secteur Nord-Batiment A - Bd Pierre Dramard, 13344 Marseille Cedex 15, France; 3 Department of Genetics, Development and Molecular Biology, School of Biology, Aristotle University, Thessaloniki, 54124 Thessaloniki, Greece; 4 Department of Classics, Stanford University, Stanford, CA 94305, USA; 5 Sorenson Molecular Genealogy Foundation, Salt Lake City, Utah, 84115, USA; 6 Génétique moléculaire de la spermatogenèse, Inserm UMR 910, Faculté de médecine, Marseille, France; 7 UMR6130, CNRS, Université Nice Sophia Antipolis, Campus Saint-Jean-d'Angély, 24 avenue des Diables Bleus, 06357 Nice Cedex 4, France; 8 Dipartimento di Genetica e Microbiologia, Università di Pavia, Pavia, Italy; 9 Department of Biology, University "Tor Vergata', Ro me 00133, Italy

## Abstract

**Background:**

The process of Greek colonization of the central and western Mediterranean during the Archaic and Classical Eras has been understudied from the perspective of population genetics. To investigate the Y chromosomal demography of Greek colonization in the western Mediterranean, Y-chromosome data consisting of 29 YSNPs and 37 YSTRs were compared from 51 subjects from Provence, 58 subjects from Smyrna and 31 subjects whose paternal ancestry derives from Asia Minor Phokaia, the ancestral embarkation port to the 6^th ^century BCE Greek colonies of Massalia (Marseilles) and Alalie (Aleria, Corsica).

**Results:**

19% of the Phokaian and 12% of the Smyrnian representatives were derived for haplogroup E-V13, characteristic of the Greek and Balkan mainland, while 4% of the Provencal, 4.6% of East Corsican and 1.6% of West Corsican samples were derived for E-V13. An admixture analysis estimated that 17% of the Y-chromosomes of Provence may be attributed to Greek colonization. Using the following putative Neolithic Anatolian lineages: J2a-DYS445 = 6, G2a-M406 and J2a1b1-M92, the data predict a 0% Neolithic contribution to Provence from Anatolia. Estimates of colonial Greek vs. indigenous Celto-Ligurian demography predict a maximum of a 10% Greek contribution, suggesting a Greek male elite-dominant input into the Iron Age Provence population.

**Conclusions:**

Given the origin of viniculture in Provence is ascribed to Massalia, these results suggest that E-V13 may trace the demographic and socio-cultural impact of Greek colonization in Mediterranean Europe, a contribution that appears to be considerably larger than that of a Neolithic pioneer colonization.

## Background

**Figure 1:**
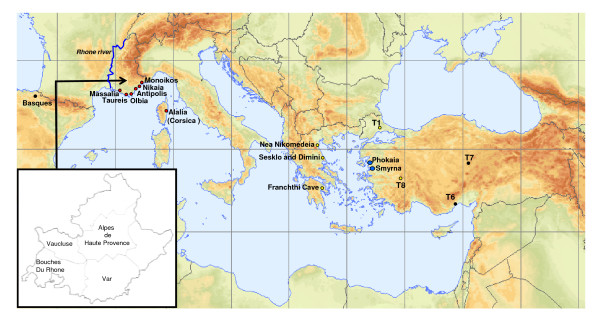
**Map showing locations of the Asia Minor Greek cities, Phokaia and Smyrna, in blue, and their western Mediterranean colonies, in red, as well as source populations studied in Greece and Turkey (in yellow)**. Putative source populations including Basques, central Anatolians, and Mediterranean Anatolians are designated with black circles. Inset shows the 4 districts in Provence from which samples were collected.

The collapse of the Late Bronze Age societies of the Eastern Mediterranean (circa 1200 BCE) led to a cascade of initial demographic retrenchment then expansion, particularly among the Phoenicians of the coastal Levant and the Greeks of the Aegean Sea [1]. Both the Greeks and Phoenicians established a set of partitioned colonies along the coast of Mediterranean Europe and North Africa and engaged in extensive trade of a variety of goods including tin and other minerals, wine and olive oil [2]. The Greeks, at the beginning of the 1^st ^millennium BCE founded cities along the Asia Minor (Anatolian) coast, divided into the Aeolian cities of northwest Anatolia, the Ionian cities of central western Anatolia and the Dorian cities of southwest Anatolia [1,3]. Although the Greek colonies of Magna Graecia of southern Italy and Sicily were established from a mixture of predominantly Dorian cities of the Aegean, the Peloponnesus and central Greece, the historical attestation of the Greek colonization of the western Mediterranean coastal regions of Provence, Spain and Corsica indicates a dominant influence from the Ionian city of Phokaia (AKA Focia, Phocaea) (Figure 1) [4]. The Phokaian Greeks founded the city of Massalia circa 600 BCE at the location of the present city of Marseille and Alalie circa 560 BCE on the eastern coast of Corsica [4]. Here the Phokaians encountered and interacted with the indigenous Celto-Ligurian populations, as evidenced by large caches of wine amphora, which the local tribes distributed along the Rhone River and the Mediterranean coast [5].

Few genetic studies have explored the Greek contribution to the modern populations of Italy and France. A recent study of Y-chromosome haplogroups in a Sicilian population [6] showed a major impact of presumptive Greek immigration to the island estimated by admixture analysis to be about 37% using a localized Balkan/Greek marker E-V13. This level of admixture was higher than that predicted by classical demographic studies. Previous Y-chromosome genetic studies of Phoenician colonization have demonstrated that haplogroup J2 frequency was amplified in regions containing the Phoenician colonies of Iberia and North Africa in comparison to areas not containing Phoenician colonies [7]. However, these studies did not address the role of either Greek colonization or early Neolithic colonization of Western Europe.

Y-chromosome studies have investigated the contribution of various Y haplogroups to the spread of farming from the Near East to Europe [8-10]. Haplogroup J2 frequency has been correlated with aspects of the symbolic material culture of the Neolithic in Europe and the Near East (painted pottery and ceramic figurines) [11] and sub-Haplogroups of J2 have also been associated with the Neolithic colonization of mainland Greece, Crete and southern Italy [12]. On the other hand, E-V13 appears to have originated in Greece or the southern Balkans [13,14] and then spread to Sicily at high frequencies with the Greek colonization of the island. E-V13 is also found at low frequencies on the Anatolian mainland [13] and thus may be useful in teasing apart the relative contributions of Greek colonization (E-V13) from Early Neolithic colonization (J2) to Western Europe. In this report, a sampling of individuals whose ancestry traces to the Ionian Greek city of Phokaia will be compared through Y-chromosome genotyping to samples from the Aeolian/Ionian city of Smyrna and a set of samples from Provence. These data will reveal genetic patterning characteristic of 1) the Ionian foundation of Phokaia versus the Aeolian/Ionian foundation of Smyrna. 2) the relative Y chromosome contributions of Phokaian Greeks and local Anatolian/Neolithic and/or central Anatolian populations in these two Asia Minor Greek city-states and 3) the contribution of Greek and/or Neolithic Y-chromosomes to the demographic pattern of Provence.

## Results

**Figure 2:**
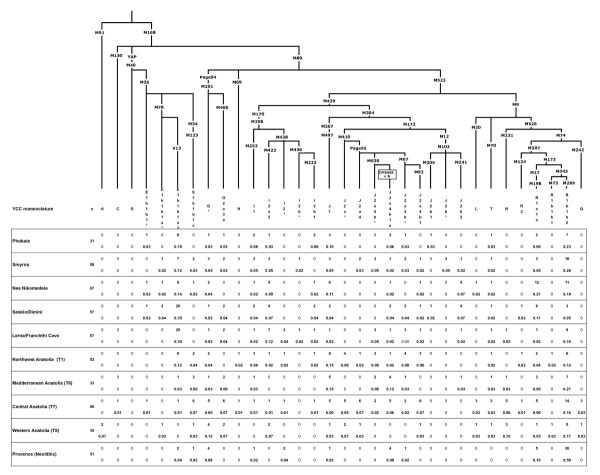
**Phylogenetic relationships and Y-chromosome haplogroup absolute and relative frequencies in the following various populations: Anatolian Greeks, mainland Greeks, four regions in Turkey and Provence near known Neolithic settlements**.

The phylogenetic relationships and haplogroup frequencies for the data from the two sites in Asia Minor: Phokaia and Smyrna, three mainland Greek sites, the four regions from Turkey and the Neolithic sites in Provence are given in Figure 2. Phokaia and Smyrna have just subtle differences in their haplogroup composition. The dominant haplogroups in both Phokaia and Smyrna are E-V13 (19.4% and 12.1%) and R1b-M269 (22.6% and 27.8%) respectfully. In addition, J2a is also common, attaining a frequency of 9.7% in Phokaia and 15.5% in Smyrna. This high frequency of haplogroup J2a-Page55 (formerly DYS413≤ 18) in Smyrna is characteristic of non-Greek Anatolia. Table 1 describes populations analyzed in this study. The AMOVA (Table 2) showed no significant distinction between Phokaia and Smyrna, whereas Smyrna was significantly differentiated from central Anatolia and Phokaia from western Anatolia. Smyrna also differed from both the Sesklo/Dimini samples from Thessaly and the Lerna/Franchthi Cave samples from the Peloponnese. The AMOVA analysis demonstrated that both language/religion and geography discriminated the sample groups (Table 3).

**Table 1:** Populations used for AMOVA, MDS and Admixture Analyses

Population	N	Geography	Language	Reference	Admixture
Phokaia	31	West Asia Minor	Greek	This Study	Anatolian Greek source

Smyrna	58	West Asia Minor	Greek	This Study	Anatolian Greek source

Nea Nikomedeia	57	Greek Macedonia	Greek	Ref. 12	

Sesklo/Dimini	57	Greek Thessaly	Greek	Ref. 12	

Lerna/Franchthi	57	Greek Peloponnesus	Greek	Ref. 12	

T1	52	Northwest Anatolia	Turkish	Ref. 24	

T6	33	Mediterranean Anatolia	Turkish	Ref. 24	Anatolian Neolithic source

T7	90	Central Anatolia	Turkish	Ref. 24	Anatolian Neolithic Source

T8	30	West Anatolia	Turkish	Ref. 24	

Provence (Neolithic)	51	France-Provence/Monaco	French	This Study	

Provence (surname based)	368	France-Provence	French	This Study	

Corsica	323	France-Corsica	Corsican	This Study	

Basque	116	Spain-Basque	Basque	Ref. 27	Indigenous source

**Table 2:** Fst analysis of haplogroup frequency

	Phokaia	Smyrna	NN	SD	LF	T1	T6	T7	T8
Phokaia	0.00000								
Smyrna	-0.00900	0.00000							
NN	0.00011	0.01120	0.00000						
SD	0.01592	0.04912*	0.03055*	0.00000					
LF	0.01021	0.03003*	0.04128*	0.00167	0.00000				
T1	0.00003	0.01101	0.01955*	0.03895*	0.03794*	0.00000			
T6	-0.00012	0.00280	0.01239	0.06960*	0.06762*	0.00372	0.00000		
T7	0.01667	0.01306*	0.02857*	0.07033*	0.06740*	0.00861*	-0.00035*	0.00000	
T8	0.02113*	0.01181	0.02940*	0.07606*	0.06794*	0.01580	0.01118	-0.00778	0.00000

*Significant Fst P < 0.05

Notes: NN = Greek Nea Nikomedeia, SD = Greek Sesklo/Dimini, LF = Greek Lerna/Franchthi Cave, T1 = northwest Anatolia, T6 = Mediterranean Anatolia, T7 = central Anatolia, T8 = western Anatolia

**Table 3:** AMOVA results according to language and geography

Classification	% Var. among groups (F_CT_)	% Var. among populations within groups (F_SC_)	% Var. within populations (F_ST_)
Geography	2.72* (0.027)	1.24** (0.013)	96.04 (0.040)
Language	2.33* (0.023)	1.38** (0.014)	96.29 0.037)

* P < 0.025

** P < 0.001

Notes: Geography = mainland Greece vs Turkey; language = Greek vs Turkish

**Figure 3:**
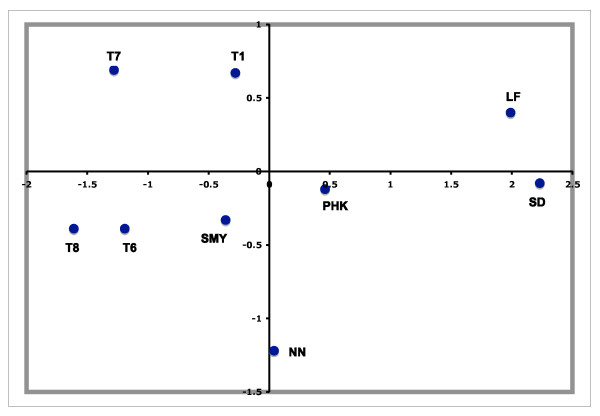
**MDS plot of Asia Minor Greek, mainland Greek and Turkish samples**. They are based on Fst of haplogroup frequencies. For the MDS analysis R^2 ^= 0.95 and Stress = 0.10. Population codes are: T1 = northwest Anatolia, T6 = Mediterranean Anatolia, T7 = central Anatolia, T8 = western Anatolia, SMY = Smyrna, PHK = Phokaia, NN = Nea Nikomedeia, LF = Lerna/Franchthi Cave, SD = Sesklo/Dimini.

MDS analyses show that both mainland Greek and Phokaia separate from the Turkish samples while Smyrna positions between mainland Greeks and the Turks (Figure 3). Since the Phokaian and Smyrnian samples could not be distinguished from each other in terms of Fst, they were aggregated for the subsequent admixture analyses.

The dominant haplogroup of Provence is R1b-M269 at 58.8% (Figure 2). Also found in Provence is haplogroup E-V13 (3.9%) and J2a-DYS445 = 6 (7.8%). All the V13 derived samples are from western Provence along the Rhone, while all the J2a-DYS445 = 6 are from Var in eastern Provence.

**Figure 4:**
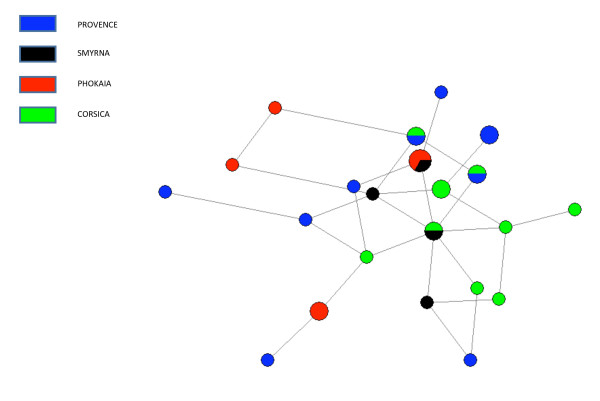
**Diversity of YSTR haplotypes belonging to haplogroup E-V13**. Reduced median network constructed from the following eight loci: DYS19, 389I, 389B, 390, 391, 392, 393 and 439. The area of each node (circle) is proportion of samples with the same haplotype and the length of each edge is proportional each mutational step.

The admixture analysis (Table 4) indicates a high level of indigenous Basque admixture throughout Provence (70-90%). Also detected is a 17% contribution of Greek Phokaia/Smyrna and a 0% Neolithic (central Anatolian and/or Mediterranean Anatolian) contribution to the Neolithic sites and surname based Provence samples. Investigating the apportionment of the pooled data from Neolithic sites and surname based into eastern Provence (n = 127) and western Provence (n = 292) the data showed a 12% Greek component and an 18% Neolithic component to eastern Provence, while attesting a 19% Greek component and a 0% Neolithic component to Western Provence. This does not exclude other sources of early Neolithic demographic episodes to Provence such as the radiation of R1b-269 sub lineages [15]. Of the 323 Y-chromosomes studied in Corsica 4.6% were derived at E-V13 in east Corsica and 1.6% in west Corsica. The network plot of eight V13 YSTRs for Provence, Corsica, Smyrna and Phokaia (Figure 4, Additional file 1: Supplemental Table S1) showed haplotype sharing among the E-V13 representatives. Coalescent times for E-V13 in Corsica, Greek Anatolia and Provence are presented in Additional file 2: Supplemental Table S2. The mean ages are oldest in Greek Anatolia consistent with it being a source population. The values should be viewed as upper bounds. The ages for Corsica and Provence exceed the founding dates from the archeological record of the Greek colonies in the Mediterranean. This discordance is most likely a consequence of multiple E-V13 founders inflating the variance.

**Table 4:** Admixture proportions (mY).

	Parental populations		
	
Hybrid Populations	Greek mY1 (SE)	Indigenous (Basque) mY2 (SE)	Neolithic (Central/Mediterranean Anatolian) mY3 (SE)
Provence (surname)	0.17 (0.15)	0.88 (0.10)	0.0 (0.13)
Neolithic Provence	0.17 (0.20)	0.82 (0.21)	0.01 (0.18)
East Provence	0.12 (0.14)	0.7 (0.16)	0.18 (0.16)
West Provence	0.19 (0.17)	0.91 (0.12)	0.0 (0.14)

Sample size used for each region: for Provence with surname criteria (n = 368), Neolithic Provence (n = 51), East Provence (n = 127), West Provence (n = 292) based on Anatolian Greek (n = 89), indigenous Basques (n = 116) and Anatolian Neolithic parental populations T6 + T7 (n = 123).

## Discussion

This study presents the first genetic data on those Greeks whose ancestry traces to western Anatolia before the 1923 exchange with Turkey. The two sites: Phokaia and Smyrna have a long established historical record and represent somewhat different Archaic Greek dialects and regions. Archaic Smyrna, a small polis of approximately 6 hectares in size, perhaps containing 700 individuals, was initially Aeolic with a subsequent immigration of Ionic Greeks from nearby Kolophon [16,17]. Phokaia was a larger Ionic city-state (50 hectares), containing an estimated 6000 individuals including its surrounding *chora*, its agricultural territory [3,16]. Smyrna, on the other hand, being a smaller polis, may show evidence of indigenous Anatolian admixture likely from neighbouring Lydia [17] with higher frequencies of J2a-Page55 derived chromosomes.

The frequency of J2a-DYS445 = 6 in Phokaia (6.5%) is comparable to that of central Anatolia (5.5%). Interestingly, the Anatolian Greek samples derived for J2a with DYS445 = 6 have DYS391 = 9 repeats, while samples from central Anatolia and Antalya in Mediterranean Anatolia and Crete, either are equally mixed with DYS391 = 9/10 or dominated by J2a-DYS445 = 6 with DYS391 = 10 or more repeats. The similar frequencies of J2a-DYS445 = 6 in the Greek city-state and Anatolia make the marker less useful for detecting a pure Neolithic component in other regions; however, the separation by DYS391 offers some utility in teasing apart the relevant components.

In France, Massalia was the unique initial Greek colony founded by the Phokaians circa 600 BCE [4]. The initial colony was small, likely 12 hectares in area, but rapidly expanded during the following century to 40 hectares [5]. Thus, its initial population may have numbered 1000 to 1500 rapidly growing to 5000 people including its small hinterland *chora*, later cultivated in large part with vineyards. In contrast, the departments of Var, Vaucluse and Bouche-du-Rhone contain an area of 14,000 square kilometers. During the Roman period, according to Beloch [18] who estimates a density of 10 individuals per square km in northern Italy, the population might have numbered 140,000. Even with an earlier 600 BCE reduction in population density around Massalia, it is probable that the indigenous Ligurians may have numbered at least 50,000. This would have yielded a maximum of 10% Greek input to Provence, much lower than the estimated 20% Y-chromosome input. However, this increase in Y-chromosome admixture from Greece is in accord with the recent results from Sicily, which estimated a 37% Greece input, in accordance with the demographic estimate of [18,19]. We acknowledge that population history of Provence has been influenced by additional demographic events besides the Neolithic and Greek colonization events. One potential confound is the impact of the Roman Empire. However in other regions well known to have been settled by Romans, e.g. England, southern Spain, Morocco and Sardinia, the frequency of E-V13 ranges from zero to 1% [13]. The impact of Phoenicians is minimal since the frequency of E-V13 in Lebanon is zero out of 42 samples (unpublished results, OS). Thus the presence of E-V13 in the western Mediterranean is most likely driven by Greek colonists. Interestingly the female input, estimated using mtDNA data may be minimal in Provence. One mtDNA study of Var, showed a negligible Neolithic (Near Eastern and hence Greek) component to the mtDNA distribution of Var [20]. Results from a single locus like the Y chromosome phylogeny must be interpreted cautiously since haplogroup designation and population are not absolutely equivalent. In addition founder effects, sex-biased reproduction, sexual selection can skew the interpretation of a population's history.

The Greeks of Massalia, between 500 BCE and 300 BCE, conquered a vast nearby area and set up satellite trading posts, settlements and forts. These sites included Monoikos (Monaco), Nikaia (Nice), Antipolis (Antibes), Olbia and Tauroeis [4,5]. The Greeks from Massalia also engaged in a major trading network along the Mediterranean coast and up the Rhone evidenced by Massaliote wine amphora and other ceramics [5]. Our data are consistent with a male-mediated asymmetric gene flow into the indigenous Celto-Ligurian populations of Iron Age Provence due to possibly differential mating practices, elite dominance or enslavement.

The island of Corsica contains E-V13 Y-chromosomes, particularly in the eastern portion of the island at a frequency of 4.6%. Eastern Corsica was the site of a major Phokaian colony, Alalie, and the E-V13 network pattern suggests overlap among the regions studied. On the other hand, using J2a-DYS445 = 6, G-M406 and J2a-M92, we detected a Neolithic (Anatolian), impact on the demography of east Provence. This may be a slight overestimate, since no J2a-M92 or G-M406 derived chromosomes were found in the Provence samples. That said, the predominant region in which J2a-DYS445 = 6 lineages are present is Var, situated near initial Neolithic impressed ware sites [21]. West of Var, J2a-DYS445 = 6 frequency drops off precipitously suggesting the demographic impact of Neolithic colonists from Anatolia does exceed beyond this region. The western districts of Vaucluse and Bouches du Rhônes contains Mesolithic sites and later cardial Neolithic package [21]. The high level of indigenous Basque admixture in Provence is consistent of a model of the cultural diffusion of agriculture. The lack of Y-chromosome Neolithic markers in west Provence suggests that the subsequent cardial Neolithic may reflect a cultural adoption of farming in this area.

## Conclusion

The Greeks from both mainland Greece and Anatolia made a major contribution to the development of western European culture through their Mediterranean colonies (Italy, France, and Spain) during the Iron Age. Haplogroup E-V13 may trace the movement of the Ionian Greeks to key areas of France and Corsica that introduced viniculture to Western Europe [22]. Further studies will help elucidate the relative contribution of the Greek and Neolithic migrations in other areas of the western Mediterranean.

## Methods

Our population samples included a total of 89 male subjects, currently living in Greece, who trace their grand-paternal ancestry to either the area near Phokaia (n = 31) or Smyrna (n = 58) prior to the 1923 Exchange of Lausanne. In addition 323 males living throughout Corsica who trace their paternal ancestry to the island, and 51 subjects from villages near Neolithic sites in Provence who trace their grand-paternal ancestry to Provence and the Principality of Monaco were also studied. A total of 23 of the subjects from Provence villages were from the western departments of Provence: Vaucluse and Bouche-du- Rhone, while 28 apportioned to the eastern departments: Var and Alps-de-Haute-Provence or to Monaco. Regarding the new samples introduced in this study, the Anatolian Greek component was approved by the IRB of Aristotle University, Thessaloniki, Greece. The French samples were approved by the French Committee for the Protection of Persons in Biomedical Research (CCPPRB) and the entire French collection were also declared to and approved by the French Ministry of Higher Education and Research. All subjects gave their informed consent to participate in the study. The location of the Anatolian Greek, mainland Greek, Turkish and Basques samples are shown in Figure 1. In addition the locations of Massalia and its trading posts and the Greek city of Alalie in Corsica are indicated. Additionally, a description of populations analyzed in this study is summarized in Table 1.

All 89 samples from Anatolian Greeks were genotyped using 29 Y-chromosome binary polymorphisms in a sequential manner using Y tree branching patterns to infer upstream haplogroup status. The following binary markers were genotyped: YAP, M35, V13, M78, M123, M34, M102, M9, M70, M74, M198, M269, M304, M497, M12, M241, M205, Page55, M67, M92, M530, M258, M253, M436, M223, P37.2, M423, M406, and M530. M497, Page55, Page94, and M530 are newly listed SNPs whose specifications are listed in Additional file 3: Supplemental Table S3. Binary marker genotyping was done by RFLP assay, DHPLC or direct sequencing. Each of the Phokaia and Smyrna samples were typed at 37 YSTRs listed in Additional file 4: Supplemental Table S4.

The 51 samples from areas near Neolithic sites in Provence had derived alleles for the following markers: V13, M34, Page94, M253, M438, M497, M530, M67, M198 and M269. In order to compare the selected 51 Neolithic samples to a larger Provence set, 368 subjects from the departments of Provence: Var (n = 68), Bouche-du-Rhone (n = 209), Vaucluse (n = 60) and Alps-de-Haute-Provence (n = 31) whose surname was blindly determined to be of French origin, were genotyped for E-V13, M406, Page94, M423, M269 and all the following J-lineages: M304, Page55, M267, M12, M410, M67, M530 and M92 as well as ten YSTRs for the J and E-V13 derived samples. DYS445 was typed in M530 derived samples (Schrack B.E., Athey T.W., Wilson J.F., 2006, The American Society of Human Genetics. Abstract). The 323 samples from Corsica were only typed for E-V13.

An AMOVA [23] was performed using Arlequin 2000 [24] to test the population affinities of the two Anatolian Greek samples to three mainland Greek samples (Nea Nikomedeia, Sesklo/Dimini, Lerna/Franchthi Cave), and four regions of Turkey (western Aegean, Marmara, central Anatolia and Mediterranean Turkey) [25]. Furthermore a Multidimensional Scaling analysis (MDS) (SPSS 18.0) was performed using the Fst measure as a distance metric across the 9 populations. An AMOVA comparing the effects of geography (Asia Minor vs. Mainland Greece) and religion/language (Christian/Greek vs. Muslim/Turkish) was also calculated using these 9 populations.

To analyze the impact of the attested Greek colonization of Provence, an admixture analysis [26] was conducted using a Basque population (n = 116) [27] as an indigenous (non-Neolithic pre-Greek) source population and the Phokaia/Smyrna data as the Greek colonizing source represented by E-V13 frequency. As a signal of putative Neolithic immigration to Provence, central Anatolian and Mediterranean Turkey data [25] were used. Specifically the following markers M92, M406 and J2a-(DYS445 = 6) were chosen as indicative of Neolithic ancestry. The frequencies of M92 and J2a-(DYS445 = 6) in the Basque population were estimated from their YSTR pattern [27]. In order to assess the degree of E-V13 affinity, a 8 loci YSTR network using Phokaia, Smyrna, Provence and Corsica samples was constructed [28]. Networks were constructed by the median joining method using Network 4.5.0.2, where ε = 0 and microsatellite loci were weighted proportionally to the inverse of the repeat variance observed in each haplogroup[29]. Coalescent times for E-V13 based on the following 8 loci DYS19, DYS389I, DYS389II, DYS390, DYS391, DYS392, DYS393 and DYS439 were computed using the methodology of Zhivotovsky et al.[30] as modified according to Sengupta et al.[31]. A microsatellite evolutionary effective mutation rate of 6.9 × 10^-4 ^per 25 years was used [30].

## Authors' contributions

RJK conceived of the study, did its design and wrote the manuscript. JDC, AK, CT, NMM, AAL, AE, MM did molecular genetics analyses. Authors CT, AK, OS and AN contributed samples and genetic data. WS, DB and PAU participated in the study design and contributed to the text. JC participated in its design and coordination the overall project. All authors read, comment and approved the final manuscript..

All authors declare no interest conflict.

## Supplementary Material

Additional file 1 **Supplemental Table S1**. E-V13 STR haplotypes. Click here for file

Additonal file 2 **Supplemental Table S2**. Coalescence Times for haplogroup E-V13. Click here for file

Additional file 3 **Supplemental Table S3: Description of new Y SNPs**. Click here for file

Additional file 4 **Supplemental Table S4**. Binary marker and YSTR 37 loci haplotypes for Anatolian Greek population samples. Click here for file
